# Exploring perspectives of interest‐holders on the use of health and genomic data from deceased participants in research: An updated systematic review

**DOI:** 10.1002/jgc4.70186

**Published:** 2026-03-02

**Authors:** Katarzyna Kucharska, Ahmed Hassan Ali, Frank Moriarty

**Affiliations:** ^1^ School of Pharmacy and Biomolecular Sciences RCSI University of Medicine and Health Sciences Dublin Ireland

**Keywords:** data‐sharing, ethics, genetic data, health data, incidental findings, post‐mortem, privacy

## Abstract

The use of research biobanks and databases often involves prolonged storage of data, meaning that an increasing amount of deceased participants' data is being used in research. Research participants are not always informed of the intent to continue using their data post‐mortem, and using such data affects the privacy of decedents and their surviving relatives. It is therefore important to assess the perspectives of interest‐holders in this respect, considering the rapid progress of big‐data technologies, new privacy regulations in the EU and unprecedented data sharing during the COVID‐19 pandemic. This paper aimed to update a systematic review by Bak et al., to investigate the views of interest‐holders on post‐mortem data sharing in research. This systematic review followed the same search strategy and inclusion criteria as the previous review, focusing on new empirical evidence on the views of interest‐holders regarding the post‐mortem sharing or re‐use of genetic or health data of research participants, from studies published in 2019–2025. It is reported based on the Preferred Reporting Items for Systematic Reviews and Meta‐Analysis (PRIMSA) statement. Findings of included studies were narratively synthesized. The updated systematic review identified seven studies involving 2151 participants, which were of high quality. The main themes of these studies related to perceived acceptability of post‐mortem data sharing, aspects of consent (including broad consent), sharing clinical findings with relatives, and barriers and facilitators to data sharing. The findings illustrate that post‐mortem genetic and health‐related data use remains a relatively under‐explored subject, with evident gaps in legislation and guidance.

**What is known about this topic**

A previous review on this topic has shown that interest‐holders are divided about aspects of managing genetic and health data of deceased research participants. The situation is further complicated by varying laws and guidelines on the management of post‐mortem data.

**What this paper adds to the topic**

This paper synthesizes perspectives from new interest‐holders from broader fields of research. It provides insight into their perspectives on posthumous data sharing following General Data Protection Regulation (GDPR) implementation and the COVID‐19 pandemic.

## 1 INTRODUCTION

The surging use of databases and biobanks which store and utilize data for long periods of time, coupled with aging populations means that an increasing amount of deceased participants' information is being re‐used in research (Bak et al., 2020; Harbinja & Pearce, 2020; Krutzinna et al., 2019; Ursin & Stuifbergen, 2018). Initiatives to establish posthumous medical data donation systems have emerged to utilize the genetic data and other health‐related data of decedents for the benefit of future generations (Krutzinna et al., 2019). For the purpose of this paper, genetic data are considered to be any data derived from analysis of the genome; other health‐related data include but is not limited to electronic health records, claims data, fitness tracking data, etc. Although these systems and technologies can have a positive impact, they also pose new threats to ante‐mortem and post‐mortem privacy, and the latter remains largely unregulated. While GDPR in Europe classifies genetic and health‐related data as special categories of personal data which demand additional protection, GDPR does not apply to deceased persons' data (Terletska, 2023). At the same time, research participants are not always informed of the intent to continue using their data post‐mortem (Bak et al., 2020; Hänold et al., 2017; McGrath et al., 2024; Ursin & Stuifbergen, 2018). Guidance regarding disclosure of clinical findings from deceased participant data which may be clinically relevant to relatives is also sparse (Bak et al., 2020; Hänold et al., 2017; Terletska, 2023; Ursin & Stuifbergen, 2018; Wolf et al., 2015). This lack of regulation and ethical guidelines leads to confusion and variable approaches amongst research centers and biobanks in handling deceased participants' data (Bak et al., 2023).

Future technological advancements may present uses of participants' data that were not considered while giving initial consent to participate in research. As participants cannot feasibly predict how their data may be used in the future, the validity of consent is important to explore when examining the acceptability of using a participant's data post‐mortem (Bak et al., 2020; Fabry & Alfano, 2024; Krutzinna et al., 2019; Pearce, 2022). Many long‐term studies and data repositories leverage once‐off broad consent to facilitate the utilization of data for multiple research purposes. However, it has been suggested that participant preferences regarding data sharing can change over time (Ho et al., 2022; Pacyna et al., 2020). Thus, the preferences specified by the participant at the time of initial consent may not be aligned with their preferences at the time of death, making once‐off consent unsuitable (Ho et al., 2022; Pacyna et al., 2020). Continued research using deceased participants' data could also have consequences for living relatives. Data which were previously anonymized or pseudonymized may become identifiable as more information about the individual becomes publicly available (Carmi, 2020; Sweeney et al., 2013). Even if a participant expresses a wish for their information to be used for post‐mortem research, their surviving relatives could have objections to continued biobank storage or dissemination of health data on the grounds of their own privacy (Bak et al., 2020, 2021; Bak & Willems, 2022; Gordon et al., 2019; Smolensky, 2009). In such cases, should the preferences of the living or dead persist and how should researchers and genetic counselors navigate conflicts?

The situation is further complicated by conflicting views amongst interest‐holders. A systematic review by Bak et al. (2020) revealed that researchers and the public disagree regarding some aspects of post‐mortem data control, which may pose challenges in developing policies in this space (Bak et al., 2020). Given the sensitivity of health and genetic data and the potential impacts on living and deceased individuals, it is important to explore this dissensus further and ensure that the concerns of interest‐holders are addressed before implementing new guidelines, systems or policies for biobanks and other research databases to tackle this issue. As stated by the Nuffield Council on Bioethics “the principle of participation requires decision makers not merely to imagine how people with morally relevant interests ought to expect data to be used but to take steps to discover how they do, in fact, expect data to be used and to engage with those expectations” (Nuffield Council on Bioethics, 2015).

Developments in recent years, such as the COVID‐19 pandemic and growth of AI and real‐world data use, have changed the environment for health information sharing. As new technology makes its way into our lives, our views on privacy, death, and societal expectations of posthumous rights could change (Gerdon et al., 2021; Moulaei et al., 2023; Nuffield Council on Bioethics, 2023; Rubinger et al., 2023). Therefore, this systematic review aims to explore the newly available evidence on perspectives of interest‐holders regarding the continued use of post‐mortem health and genetic data in research from 2019 to 2024, updating the previous Bak et al. (2020) systematic review.

## 2 METHODS

An update of the previous systematic review by Bak et al. (2020) was deemed appropriate based on the checklist on when and how to update systematic review (Garner et al., 2016). As per the checklist, the authors deemed that the published review addresses a current question and were aware that new studies have been published in the field. Given changes in the information sharing landscape since the last review including data sharing during the COVID‐19 pandemic and the increasing use of AI, the authors theorized that updating the review may bring new, insights regarding the perspectives of interest‐holders around continued use of data post‐mortem in research (Gerdon et al., 2021; Moulaei et al., 2023; Nuffield Council on Bioethics, 2023; Rubinger et al., 2023). The methods align with the recommendations outlined in the Cochrane Handbook of Systematic Reviews (Higgins et al., 2023), and the review is reported based on the Preferred Reporting Items for Systematic Reviews and Meta‐Analysis (PRIMSA) statement (Page et al., 2021).

### 2.1 Search strategy

The search strategy defined in Bak et al. (2020) were followed to search articles in the PubMed/Medline, EMBASE, Web of Science, and CINAHL databases. The search strategy involved the inclusion of database‐specific subject headings as well as free‐text terms focusing on the following concepts: “post‐mortem, health‐related and genetic data, research ethics, stakeholders, preferences” (Bak et al., 2020). As per the previous review, the search criteria covered all types of health‐related, taking an exploratory approach to health‐related data in this review, without confinement to a particular type of data (Bak et al., 2020). Synonyms and sub‐topics of these main concepts were also included. The search strategy is illustrated in Table 1 (the full search strategy is available in Tables [Link], [Link], [Link]). The search dates were set from January 1, 2019 (the previous review searched to December 31, 2018) to the date of the search (March 29, 2024). The search was repeated on October 7, 2025, to capture any new literature published since the initial search due to the time that has passed between the initial search and the completion of the manuscript. Upon completing the search, the systematic review was registered on Prospero (ID CRD42024531729) (Kucharska et al., 2024).

**TABLE 1:** Search strategy adopted from the Bak et al. (2020) systematic review.

Set#	Search string	Results
1	“Registries”[MeSH] OR “health data” OR “health information” OR “Electronic Health Records”[MeSH] OR biobank*[tiab] OR bio‐bank*[tiab] OR biorepository*[tiab] OR “Databases, Factual”[MeSH] OR registry[tiab] OR registries[tiab] OR databank*[tiab] OR genomic*[tiab] OR “genetic research”[tiab] OR genetic*[tiab] OR “individual finding*”[tiab] OR “genetic finding*”[tiab] OR “Genetics”[MeSH]	2,092,329
2	“Privacy”[MeSH] OR “Confidentiality”[MeSH] OR “Personally Identifiable Information”[MeSH] OR privacy*[tiab] OR confidential*[tiab] OR “Informed Consent”[MeSH] OR “informed consent”[tiab] OR “Ethics, Research”[MeSH] OR “Patient Rights”[MeSH] OR disclos*[tiab] OR “Information Dissemination”[MeSH] OR “Communication”[MeSH] OR communicat*[tiab] OR “Duty to Recontact”[MeSH]	870,541
3	“Patients”[MeSH] OR “Stakeholder Participation”[MeSH] OR famil*[tiab] OR relative*[tiab] OR participant*[tiab] OR population[tiab] OR public[tiab] OR community[tiab] OR societ*[tiab] OR “Research Subjects”[MeSH] OR researchers[tiab] OR institutions[tiab]	6,339,992
4	“Death”[MeSH] OR deceased[tiab] OR death*[tiab] OR departed[tiab] OR died[tiab] OR dead[tiab] OR post‐mortem[tiab] OR postmortem[tiab] OR posthumous[tiab]	1,486,639
5	opinions[tiab] OR perspectives[tiab] OR views[tiab] OR experiences[tiab] OR viewpoint*[tiab] OR willingness[tiab] OR preference*[tiab] OR attitude*[tiab] OR impact[tiab] OR choice*[tiab] OR support[tiab]	3,763,378
6	(#1 AND #2 AND #3 AND #4 AND #5 Filters: English, from 2019 to 2024)	211

*Note*: The PubMed version is shown as an example.

### 2.2 Inclusion and exclusion criteria

The inclusion and exclusion criteria from the previous systematic review were adopted (Bak et al., 2020). Studies were considered eligible for inclusion if they were quantitative or qualitative empirical studies which examined (a) post‐mortem sharing or re‐use of genetic or health data that was obtained during the participant's lifetime and/or (b) perspectives and experiences of interest‐holders regarding the process of such data sharing or re‐use. Studies were excluded if they were (a) a systematic review, (b) a descriptive article or case study without empirical data, (c) focused on the perspectives pertaining to post‐mortem use of data that were obtained after the death of the participant and/or (d) not written in English. There was no exclusion based on the characteristics of the participants or populations involved in the study, as long as it pertained to interest‐holders such as study participants, their family members, or researchers.

### 2.3 Study selection

The records were checked for duplicates using Covidence, a web‐based software platform (Veritas Health Innovation, Melbourne, Australia). Each record was independently screened for inclusion based on title and abstract by two reviewers (of KK, AHA, or FM) in Covidence software. Potentially eligible records proceeded to the second round of screening based on full text review, independently assessed by two reviewers (KK, AHA). Any disagreements at either round were discussed and resolved by consensus and with input from a third reviewer (FM).

### 2.4 Risk of bias and data extraction

A data extraction sheet was developed in Microsoft Excel with the following sections:

- Study details (title of paper, first author, journal, year, geographical location)
- Study design
- Sampling method
- Samples size and response rate
- Participant characteristics (type of interest‐holder/characteristic of note, age, ethnicity, and sex)
- Relevant outcomes/measures, that is, the quantitative and qualitative findings from each study. These related to participant attitudes, perspectives and willingness to donate health‐related data, or any other relevant component reported in the results.
- Risk of bias assessment using the Joanna Briggs Institute tools for qualitative research and for analytical cross‐sectional studies (Joanna Briggs Institute Critical Appraisal Tools). This was also used in the previous systematic review (Bak et al., 2020).

Quality appraisal and the subsequent extraction of data were carried out by a single reviewer (KK) and validated by a second reviewer (AHA). Where disagreement arose, a third reviewer's opinions were sought to reach consensus (FM).

### 2.5 Narrative synthesis

Narrative synthesis was employed to accommodate both the quantitative and qualitative study types in the review. The steps taken were as follows:

1. Familiarization with data
2. Extracting relevant data and grouping it into similar concepts
3. Comparing similarities and differences between studies within identified groupings

The synthesis was carried out by a single reviewer (KK) and validated by a second reviewer (AHA) with input from a third reviewer (FM), when needed.

## 3 RESULTS

**FIGURE 1:**
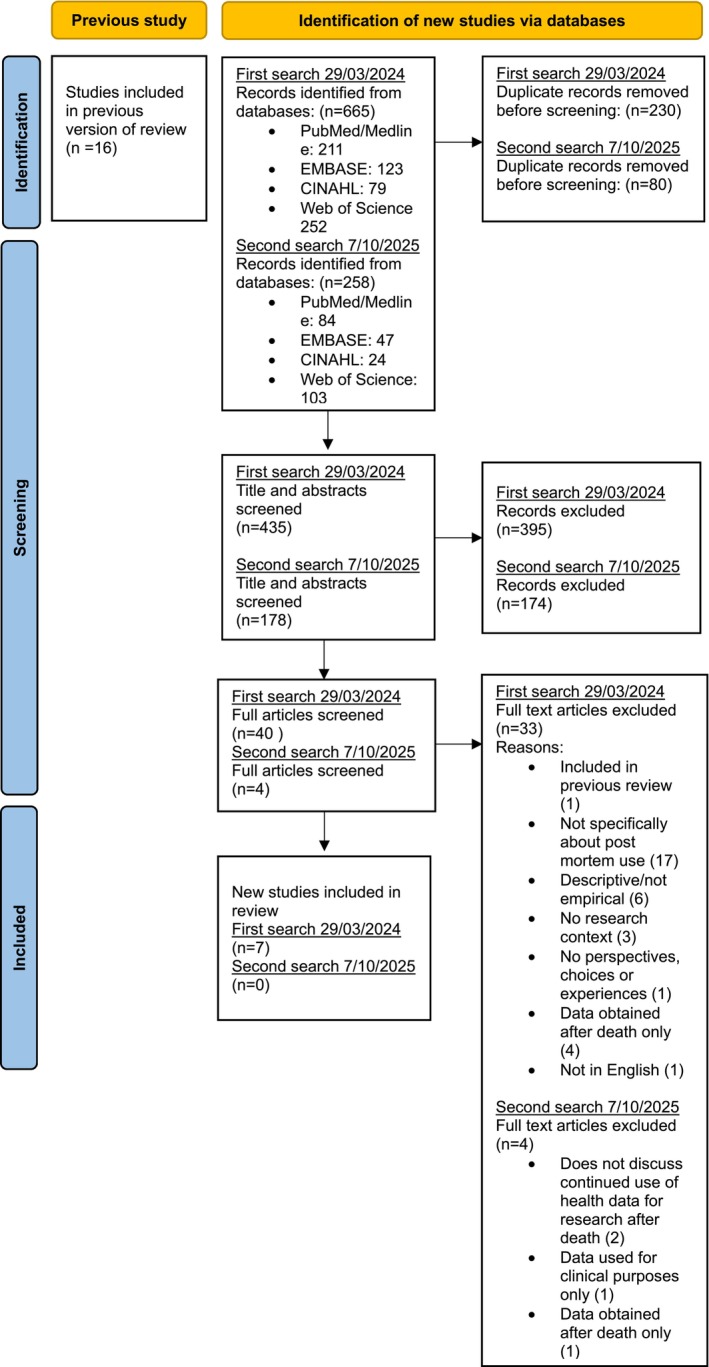
PRISMA 2020 flow diagram for updated systematic reviews, adapted from Page et al. (2021).

As illustrated by the PRISMA flow diagram adapted from Page et al. (2021) in Figure 1, 923 records were identified, with 613 remaining after deduplication. Following the initial title/abstract screening, 44 of these records qualified for full‐text review, adopting the exclusion and inclusion criteria from the previous review (Bak et al., 2020). After full‐text review, 7 studies were eligible for inclusion. All studies scored high in terms of quality appraisal, as per Table 2.

**TABLE 2:** Outcome of the assessment of studies included in the systematic review using the Joanna Briggs Institute (JBI) tools for qualitative research and for analytical cross‐sectional studies.

Study reference number	Study design	Quality appraisal	Tool utilized
Alrabadi et al. (2019)	Cross‐sectional survey	6 out of 8	JBI critical appraisal checklist for analytical cross‐sectional studies
Bak et al. (2021)	Qualitative semi‐structured interviews	9 out of 10	JBI critical appraisal checklist for qualitative research
Bak et al. (2023)	Semi‐structured interviews with selected experts, online roundtable conference where open questions were discussed	8 out of 10	JBI critical appraisal checklist for qualitative research
Lessard et al. (2022)	Cross‐sectional mixed‐method sequential convergent design, in which quantitative and qualitative data were consecutively collected and analyzed	9 out of 10	JBI critical appraisal checklist for qualitative research
Moulaei et al. (2023)	Cross‐sectional survey	7 out of 8	JBI critical appraisal checklist for analytical cross‐sectional studies
Pacyna et al. (2020)	Survey	8 out of 8	JBI critical appraisal checklist for analytical cross‐sectional studies
Seltzer et al. (2019)	Cross‐sectional survey	6 out of 8	JBI critical appraisal checklist for analytical cross‐sectional studies

Four of the included studies were survey‐based, two studies utilized semi‐structured interviews and one adopted a mixed‐methods approach. These studies were carried out in a number of geographical locations including the United States (US) (*n* = 2) (Pacyna et al., 2020; Seltzer et al., 2019), Canada (*n* = 1) (Lessard et al., 2022), Jordan (*n* = 1) (Alrabadi et al., 2019), Iran (*n* = 1) (Moulaei et al., 2023), and Europe (*n* = 2): including one study carried out in the Netherlands (Bak et al., 2021) and another one conducted across the Netherlands, Denmark, Sweden, Italy, France, Czechia, Norway, Germany (Bak et al., 2023). The retrieved articles encompassed perspectives from multiple interested parties. Four studies included the views of patients (Alrabadi et al., 2019; Bak et al., 2021; Moulaei et al., 2023; Seltzer et al., 2019), one explored perspectives of biobank donors (Pacyna et al., 2020) and one study included the views of the general public (Lessard et al., 2022). One of the studies looked at perspectives of next‐of‐kin (Bak et al., 2021) and another investigated views of researchers and ethico‐legal experts (Bak et al., 2023).

A total of 2151 participants were included in all the studies combined (the number of participants ranged from 19 to 1164). In terms of the participant characteristics, it is noted that, in two studies, over 90% of participants were white (Pacyna et al., 2020; Seltzer et al., 2019), and in three studies, the majority of participants were over 60 years of age (Bak et al., 2021; Pacyna et al., 2020; Seltzer et al., 2019). Two of the included studies discussed perspectives in relation to biological specimens and genetic data only (Lessard et al., 2022; Pacyna et al., 2020); the other five included a range of health‐related data such as electronic health records or prescription history (Alrabadi et al., 2019; Bak et al., 2021, 2023; Moulaei et al., 2023; Seltzer et al., 2019). The characteristics of participants are presented in Table 3. Key findings from each study are detailed in Table 4.

**TABLE 3:** Characteristics of participants in studies included in systematic review.

Study reference number	Country/region	Participant characteristics	Sample size (response rate)	Study design
Alrabadi et al. (2019)	Jordan	Members of the public, over 18 years of age 52% of participants were female	476 (not reported)	Cross‐sectional survey
Bak et al. (2021)	Netherlands	Dutch sudden cardiac arrest survivors who donated clinical and socio‐economic data and genetic samples to research and their next‐of‐kin 52% participants were male and 63% were 60 or older	19 (69%)	Qualitative semi‐structured interviews
Bak et al. (2023)	Europe (Netherlands, Denmark, Sweden, Italy, France, Czechia, Norway, Germany)	Sudden cardiac arrest researchers and ethical and legal experts	34 (not reported)	Semi‐structured interviews with selected experts, online roundtable conference where open questions were discussed
Lessard et al. (2022)	Canada	HIV patients Mean age of participants was 69.6 91% of participants were white 86.5% were men	48 (77% for survey, 31% for interview)	Cross‐sectional mixed‐method sequential convergent design, in which quantitative and qualitative data were consecutively collected and analyzed
Moulaei et al. (2023)	Iran	COVID‐19 patients 36.3% of patients were aged between 38 and 47	204 (43%)	Cross‐sectional survey
Pacyna et al. (2020)	US	Biobank donors Mean age of participants was 66.4 67.5% of participants were female 91% of participants were white	1164 (69.3%)	Survey
Seltzer et al. (2019)	US	Patients at an academic urban emergency department 63% of participants were African‐American 64% of participants were female	206 (35%)	Cross‐sectional survey

**TABLE 4:** Key outcomes of studies included in the systematic review.

Study reference number	Relevant outcome(s)/measure(s)	Key findings
Alrabadi et al. (2019)	Perspectives about specificity of consent with respect to post‐mortem use Factors that influence willingness to provide consent	About 80% of the participants agreed that options for the use of samples after the death of participants should be provided on the informed consent Trust in the research team, inconvenience of the re‐contact process, and the importance of biobanks were associated with participants' willingness to provide open consent Privacy and confidentiality, doubt about future use of samples, unknown storage period, and the possibility of cross‐border sample sharing were significantly associated with participants' reluctance to provide open consent
Bak et al. (2021)	Participant and next‐of‐kin perspectives on the acceptability of continued use of post‐mortem data Perspectives on sharing incidental findings with next‐of‐kin after the participant's death Opinions on the appropriate mode of consent, including post‐mortem considerations Opinions about the ability of next‐of‐kin to make data sharing decisions on behalf of the deceased Motivations for data sharing post‐mortem	All patients believed that their given consent does not expire after death; some said that they assumed the data would continue to be used post‐mortem even if this was not specified during the consent process. Next‐of‐kin were more hesitant and some felt uncomfortable by the idea that genetic data are still being used after someone passes away. One next‐of‐kin first thought it might be good to delete the data once it served its purpose, but later changed their mind expressing that science would benefit more from the data Patients and next‐of‐kin believed that when genetic data continues to be analyzed after death, it is right to inform the relatives and give them a choice on whether they wish to be informed about clinically actionable findings. Next‐of‐kin stressed that the manner of approaching should be sensitive and tactful The researchers asked whether relatives should be informed of potentially relevant genetic findings after the participant had died, even if she/he had stated the desire for this not to happen. Opinions were divided: while some thought privacy and the patient's wishes should be respected after their passing, others looked at it in terms of beneficence and found the value for relatives' health more important. Middle grounds were also suggested—providing information to relatives if asked, but stressing this is against the participant's wishes and reporting findings only if they present a serious risk to health Generally, patients agreed that if opt‐in consent is used, this could be a one‐time, broad consent (one participant preferred a “one‐time reminder” to know that their data are still being used). Some expressed that more specific or tiered consent might hamper research participation. Others took into account the administrative burden for researchers and the limiting effect on research of specific consent. Moreover, a number of interviewees would find it too burdensome to be contacted with consent requests for each separate study and some expressed the same concern for their next‐of‐kin (after the death of the participant). Others felt that there needs to be some form of control by relatives, especially for genetic or socio‐economic data that implicates them as well Mixed opinions about the ability of next‐of‐kin to make decisions for the deceased about continued data use. Some participants weren't sure what their next‐of‐kin's decision would be; “maybe the next‐of‐kin don't want the data to be used.” Others said their family would make the right decision due to having experienced a similar cardiovascular event. Some expressed that consent should be sought from family as soon as possible, others that some time should be given for the relatives to allow them to provide a “rational answer” rather than an “emotional” one Participants indicated altruistic motivations for data sharing; “even after death you can still feel useful” Some expressed that privacy legislation is too demanding; “you should just be able to do your work. It's about people's wellbeing. I think we live with too many rules for some situations, like this one”
Bak et al. (2023)	Perspectives regarding privacy concerns after death Researcher and expert opinions on disclosure of incidental findings to relatives after the participant's death Opinions about aspects of consent for posthumous use of data/samples	A risk of posthumous reputational harm was acknowledged and a potential privacy concern for relatives Researchers focused on the consequentialist harms to health when discussing data privacy, whereas ethics‐legal experts recognized that confidentiality is important to uphold respect for autonomy and safeguard public trust in science Experts noted there is an ethical responsibility, albeit not legal in most countries to disclose clinically actionable findings that indicate a serious risk to relatives of deceased participants One researcher experienced moral distress at not being able to contact the relatives of deceased patients with relevant findings due to institutional policies A number of interviewees said that if a study may give rise to actionable findings that indicate serious risk to relatives of the deceased, the relatives should be contacted as soon as possible about the research taking place Interviewees felt that regional and international differences in REC requirements on post‐mortem data sharing complicate cooperation and affect study validity. They called for an international code of conduct and more ethical and legal guidance. Experts recommended development of policy by researchers and ethico‐legal experts detailing what counts as a serious finding and install a genetics finding committee for biobanks Interviewees agreed that the purpose of research specified in consent forms can be broad Experts agreed that it would be best to inform people during their lifetime about post‐mortem data use
Lessard et al. (2022)	Perspectives of end‐of‐life HIV patients on the factors that influence data sharing for end‐of‐life research	**Quantitative** 29.7% of participants expressed concerns for physical discomfort with HIV biobanking. 16% were concerned about privacy of data in the context of biobanking “Altruistic” benefits of donation (i.e. benefiting science and society, biomedical research, or a greater cause, and giving back) were rated higher, on average, than those on “personal” benefits. 68% indicated that motivation of participation in a biobank was to help future people living with HIV. 68% wanted to help advance scientific knowledge **Qualitative** Factors that presented a barrier to data sharing included: perceiving research as potentially risky or painful (including social suffering by marginalized communities), biobanking suggesting unforeseen research with multiple risks of misuse (“some people might worry about how it is going to be used when they are gone…. will it be used ethically?”), and perceiving researchers as self‐interested. Participants were hesitant to participate “in research that is not clear in its objectives,” or that seemed profit‐oriented Factors that facilitated data sharing included; contribution to progress in health and science, an opportunity to learn more, gifting back to the community and younger generations, identifying with, or being interested in a research topic, having worked in the health system or research, approval by ethics board and the ability to discuss with unbiased and informed professionals
Moulaei et al. (2023)	Willingness of COVID‐19 patients to share health data before and after death	42.4% stated that they would donate “all health data” to health organizations and institutions after death, 30.38% would share only some health data 17% stated that they would not share health data after death and 9.8% stated that they were “not unsure” 24.5% were willing to share medication records before death and 7.84% were willing to share it after death 3.92% were willing to share electronic medical records data before death and 36.75% were willing to share it after death 0.98% were willing to share genetic data while alive and 24.99% were willing to share it post‐mortem 64% were willing to share fitness tracking data before death but only 10% after death
Pacyna et al. (2020)	The consistency of participant preferences regarding post‐mortem access to samples	40% of respondents indicated a preference regarding posthumous sample availability on the survey (T2) that was inconsistent with the preference they had expressed when they enrolled in the biobank (T1) 94% of those with inconsistent preferences shifted from a T1 preference to restrict posthumous availability of their sample to a T2 preference to make the sample available to their legal next‐of‐kin. 76.7% of those who restricted availability at T1 chose to permit availability at T2, leaving only 136 (23.3%) “consistent restrictors” who indicated a restrictive preference at both T1 and T2 Participants with inconsistent preferences indicated very strongly held preference more frequently than consistent restrictors but less frequently than consistent permitters
Seltzer et al. (2019)	Patient perspectives on sharing health data before and after death	65% agreed to share at least one digital data type listed in the survey. Participants were more willing to share digital data after death for all data types Around 50% of participants were willing to share prescription history at the time of survey and about 75% after death Around 45% of participants were willing to share electronic health records at the time of the survey and 75% after death Around 40% of participants were willing to share genetic data at the time of the survey and less than 80% after death

Four main themes of interest were identified during data synthesis, namely: acceptability of sharing data after death, aspects of consent, perspectives on sharing clinical findings with relatives, as well as barriers and facilitators to data sharing.

### 3.1 Acceptability of sharing data after death

The studies included in the review generally indicated that patients and participants support continued sharing health‐related data post‐mortem, while the opinions of next‐of‐kin were more mixed (Bak et al., 2021; Lessard et al., 2022; Moulaei et al., 2023; Seltzer et al., 2019). Interestingly, one study revelated that willingness to share data may vary depending on the type of health‐related data in question (e.g., genetic data vs. medication records) (Moulaei et al., 2023).

Two quantitative studies included in this review explored the willingness of participants to share different types of data before and after death. One study revealed that participants were more willing to share digital data after death for all health data types, including electronic health records (75%), genetic data (80%), and prescription history (75%) compared with willingness to share at the time of the survey (45%, 40%, and 50%, respectively) (Seltzer et al., 2019). Another study exploring the attitudes of COVID‐19 patients showed that 42% of participants were willing to donate all health data to health organizations for research purposes after death, while 17% were not willing to share any health data after death. Interestingly, the participants in this study were more open to sharing genetic (25%) and electronic medical records data (37%) after death, rather than while alive (1% and 4%, respectively). However, participants were less willing to share fitness tracker data (10%) and medication records (8%) post‐mortem compared with willingness to share data ante‐mortem (64% and 25%, respectively) (Moulaei et al., 2023). A mixed‐methods study which focused specifically on end‐of‐life HIV patients revealed that 81% of survey participants were willing to participate in biobanking (Lessard et al., 2022).

In terms of qualitative data, one study explored the perspectives of patients and next‐of‐kin regarding the research use of clinical and genetic data from sudden cardiac arrest (SCA) survivors, including post‐mortem aspects. All interviewees in this study believed that their consent to participate does not end after death and understood that their samples would continue to be used (even when this was not explicitly stated during the consent process). On the contrary, opinions of next‐of‐kin regarding continued use of post‐mortem were more mixed, with some feeling uneasy about continued utilization. One next‐of‐kin suggested the data should be deleted but later changed their mind on the basis of contributing the data for a “greater purpose” (Bak et al., 2021).

### 3.2 Aspects of consent

The studies included in the review indicated that participants, researchers, and ethico‐legal experts generally support the use of broad consent in research (Bak et al., 2021, 2023). Although, for some study designs, dynamic consent may be more appropriate (Pacyna et al., 2020).

If the use of data after the participants' death is foreseen in the study, experts and the public agreed that the consent form should include a disclosure and/or option regarding the use data post‐mortem (Alrabadi et al., 2019; Bak et al., 2023). Opinions about allowing next‐of‐kin to make decisions about the use of data post‐mortem appeared mixed (Bak et al., 2021).

#### 3.2.1 Appropriate mode of consent

One study revealed that 40% of participants in a US biobank changed their preference regarding posthumous access to their sample by next‐of‐kin at the time of the interview in comparison with the initial preference provided at the time of enrolment to the biobank (Pacyna et al., 2020). This indicates that dynamic consent could be more appropriate to manage participant preferences in long‐term studies. By contrast, a qualitative SCA survivor study in Europe suggested that participants mostly agreed with the appropriateness of broad consent; some expressed the view that re‐contact would be too burdensome for them, researchers, and also for their next‐of‐kin in the event of the participant's death. Participants also expressed that more dynamic forms of consent could hamper research participation and limit possible research with their data as “you cannot always know beforehand for what research it is useful.” Participant decisions on data sharing seemed to be grounded in a trust in researchers, albeit it must be noted that some struggled to distinguish between researchers and healthcare professionals involved in their clinical care. Some participants noted that relatives should have control over sensitive forms of data after the participant's death, such as genetic and socio‐economic data, given the potential implications it could have on next‐of‐kin. At the same time, the study found conflicting opinions from interest‐holders regarding the ability of next‐of‐kin to make decisions regarding data sharing on behalf of the deceased (Bak et al., 2021).

The use of broad consent was generally supported in a study exploring the views of researchers and ethico‐legal experts in the context of SCA research. One expert in the study advocated for the use of a more dynamic mode of consent that allows participants to choose how the data are shared and whether they wish to receive information about clinical findings (Bak et al., 2023).

#### 3.2.2 Specificity of informed consent

In the study exploring the views of SCA researchers and ethico‐legal experts, the respondents agreed that participants should be informed about post‐mortem data use while alive (Bak et al., 2023). Likewise 80% of public respondents in a Jordanian survey agreed that the post‐mortem use of samples should be an option specified as part of broad consent; 88% of respondents in this study also felt that consent should make provisions for participants to specify the preferred duration of storage of the sample (Alrabadi et al., 2019). On the contrary, as described above, the findings from the study focused on SCA research, revealed that participants do not consider their consent to end after death, and understood samples would continue to be used even if this is not explicitly stated on the consent form. Some participants indicated willingness to leave decisions about appropriate use and storage of data to researchers, quoting their lack of expertise and trust in the researchers as reasons behind this (Bak et al., 2021).

### 3.3 Perspectives on sharing clinical findings with relatives

A lack of harmonization in regional and institutional guidelines regarding the disclosure of genetic incidental findings to next‐of‐kin of a deceased participant was noted by researchers and ethico‐legal experts (Bak et al., 2023). Participants of one study appeared to support the disclosure of such incidental findings to next‐of‐kin when the data continue to be used post‐mortem (Bak et al., 2021). However, the sensitivity of the data and prior consent from the participant to share any clinical findings may need to be considered (Bak et al., 2021; Lessard et al., 2022).

In one qualitative study, the views of SCA researchers and ethico‐legal experts were sought regarding disclosure of genetic incidental findings after the participant's death. During the discussions, one researcher expressed moral anguish due to not being able to disclose relevant findings to the relatives of a deceased participant because of institutional policy. The experts in this study agreed that there is an ethical, albeit not a legal duty for researchers to disclose actionable findings that could pose a serious health risk to surviving family. It was suggested that where a study is being carried out using deceased participant data that could give rise to actionable findings, next‐of‐kin should be informed as soon as possible. A lack of harmonization was noted in this area, with regional differences in guidelines. The experts recommended that guidelines on what constitutes a serious genetic finding should be developed. It was also suggested that biobanks could benefit from establishing a genetic findings committee to aid with decision making (Bak et al., 2023).

In contrast to the views of researchers and ethico‐legal experts, the study investigating opinions of SCA survivors and next‐of‐kin found that disclosure of post‐mortem actionable findings to relatives was supported if such data continue to be used. Concerns were expressed about the way the results are disseminated to relatives following the participant's death, with interviewees stressing the importance of approaching family in a tactful manner and allowing them to choose if they wish to receive the results. However, opinions were divided when interviewees were asked if disclosure of genetic findings to living relatives is appropriate even if it is against the deceased participant's expressed wishes. Some expressed that the privacy of the deceased and their wishes should be respected, and others considered the benefit to surviving family members more important. Some suggested middle‐ground approaches such as disclosing information only if it poses a serious risk to the relative's health or providing the information upon request but stressing that disclosure is against the wishes of the participant (Bak et al., 2021). A Canadian study exploring the perspectives of end‐of‐life HIV patients revealed that one of the factors affecting research participation may be concerns about the disclosure of personal information or HIV status, especially to family members (Lessard et al., 2022). As such, the type and sensitivity of data may need to be considered when sharing clinical findings with relatives.

### 3.4 Barriers and facilitators to data sharing

The studies included in this review indicated that permitting the use of data for research after death is largely motivated by altruistic reasons and trust in researchers (Alrabadi et al., 2019; Bak et al., 2021; Lessard et al., 2022). On the contrary, unknown storage periods, concerns about the ethical use of data, and unclear research objectives were barriers to post‐mortem data sharing (Alrabadi et al., 2019; Lessard et al., 2022). The issue of post‐mortem privacy was acknowledged by ethico‐legal experts (Bak et al., 2023). However, studies that explored the views of participants yielded mixed views regarding privacy concerns as a barrier to data sharing (Alrabadi et al., 2019; Bak et al., 2021; Lessard et al., 2022).

#### 3.4.1 Barriers to sharing data

The Canadian study of end‐of‐life HIV research participation revealed concerns about the risks of unforeseen research “some people might worry about how it is going to be used when they are gone… will it be used ethically?” Participants also expressed hesitation of involvement “in research that is not clear in its objectives” and profit‐oriented research. Sixteen percent of surveyed participants indicated concerns about the privacy of their biobank data (Lessard et al., 2022). Similarly, the survey exploring perspectives of Jordanians on broad consent in biobanking indicated that privacy concerns and unknown storage periods were factors that influence willingness to provide consent (Alrabadi et al., 2019). However, these findings were not specific to posthumous data use.

On the contrary, in the European study exploring the perspectives of SCA survivors and their next‐of‐kin, most interviewees did not express privacy concerns about the use of their genetic and health data in research. In fact, some participants made comments regarding privacy legislation becoming “too demanding” and were worried about it inhibiting research (Bak et al., 2021).

In the study exploring perspectives of SCA researchers and ethico‐legal experts, the existence of posthumous reputational harm was acknowledged and concerns raised about privacy implications for relatives regarding sharing deceased participant data. Interestingly, when discussing privacy issues regarding the use of post‐mortem health and genetic data, researchers tended to focus more on “consequentialist harms to health,” whereas ethico‐legal expert views were generally grounded in maintaining respect for autonomy and fostering trust in the scientific community. As mentioned previously, the experts and researchers also expressed a need for more harmonized guidelines around the access and sharing of health and genetic data, as disjointed approaches hamper cooperation among researchers and could affect the validity of studies (Bak et al., 2023).

#### 3.4.2 Facilitators of data sharing

The study exploring the views of end‐of‐life care HIV patients suggested that participants were mostly motivated by altruistic reasons to participate in biobanks such as “giving back” to science and society. Some quoted the ability to have a discussion with “informed and unbiased professionals” as well as approval by an ethics board to be important factors (Lessard et al., 2022). Similarly, the European study investigating the views of SCA survivors revealed altruistic reasons for sharing health and genetic data and that “even after death you can still feel useful.” The participants seemed to be largely influenced by trust in researchers, with one specifying “if you know it's for science, I have complete trust” (Bak et al., 2021). Trust in the research team, the inconvenience of the re‐contact process, and the importance of biobanks were factors associated with participants' willingness to provide broad consent in biobanking in Jordan; however, this was not specific to post‐mortem data use (Alrabadi et al., 2019).

## 4 DISCUSSION

The findings of this systematic review mostly align and expand on those of the previous review by Bak et al., regarding acceptability of sharing data post‐mortem, disclosure of deceased participants' genetic findings to next‐of‐kin, and altruistic motivations for data sharing. This review identified new perspectives regarding the appropriate mode of consent and its specificity in the context of post‐mortem data utilization. While the previous systematic review search strategy “encompassed all health‐related data,” the 16 studies retrieved in the previous review included findings pertaining to genetic data only. More recent literature identified in this review revealed new findings on broader health data, including electronic health data, prescription histories, and fitness tracking data of decedents. Furthermore, this review elucidated participant perspectives from a broader variety of fields including research involving SCA, COVID‐19, and HIV patients, compared with the initial review which mostly identified studies involving cancer research (Bak et al., 2020, 2021; Moulaei et al., 2023; Seltzer et al., 2019).

This review presents further, albeit limited evidence to support the acceptability of continued use of post‐mortem data in research (Bak et al., 2021; Moulaei et al., 2023; Seltzer et al., 2019). Two studies demonstrated that participants seem to be more open to share genetic data and certain types of health data post‐mortem, rather than while alive (Moulaei et al., 2023; Seltzer et al., 2019) These findings could be considered to support research policies which facilitate continued posthumous data use.

The opinions of next‐of‐kin regarding continued use of participant data post‐mortem in this review appeared mixed (Bak et al., 2021). This contrasts with the findings from the previous review, which showed high support of next‐of‐kin for continued post‐mortem use of data. However, the majority of the studies included in the previous review concerned the views of parents, who provided initial consent for the use of their children's data in research (Bak et al., 2020). By contrast, the population of the study in this review comprised spouses and blood relatives who likely did not have this initial decision‐making capacity. Thus, they may lack insight into the preferences and values of the participant leading them to choose a more conservative approach for data handling. This review indicates that participants are aware of this potential disconnect in opinions between themselves and next‐of‐kin, and some were not confident in their relative's ability to effectively manage data sharing preferences on their behalf after death (Bak et al., 2020, 2021; de Man et al., 2023).

The findings of studies included in this review demonstrated that researchers, experts, and the public believe that consent forms should include a disclosure regarding the post‐mortem use of data (Alrabadi et al., 2019; Bak et al., 2023). The findings from the previous review also indicated that most institutional review board (IRB) chairs, researchers, and participants support such disclosure (Bak et al., 2020). The OECD Guideline on Human Biobanks and Genetic Research Databases recommends that biobanks and genetic databases should have a policy to “address the situation where participants become legally incapacitated or die” (OECD, 2009). However, evidence indicates that this is not done in practice, perhaps suggesting a need for more robust local guidelines and/or legislation (Bak et al., 2020; Ursin & Stuifbergen, 2018). This may be particularly important to consider for studies which anticipate storing samples and/or data indefinitely.

The previous systematic review showed conflicting perspectives between lay people, researchers, and IRB chairs on sharing incidental findings with relatives of deceased participants. It showed that the majority (between 76.5% and 98%) of research participants, researchers, and family members supported such disclosure, in contrast with only half (45%–51%) of IRB chairs (Bak et al., 2020). This review did not include any studies that facilitate further quantitative comparison, but the qualitative studies focused on SCA research seemed to show general support amongst researchers, ethico‐legal experts, next‐of‐kin, and participants to share genetic findings post‐mortem. While an ethical responsibility to disclose clinically relevant information seemed to be acknowledged, it is not reflected by the laws and policies in many jurisdictions (Bak et al., 2021, 2023). The EU Data Protection Supervisor noted a need for harmonization in this area (Wolf et al., 2015). The Public and Professional Committee of the European Society of Human Genetics acknowledged the need to update professional guidance and suggested that plans for disclosure of genetic findings post‐mortem should be included in the design of sample collection (Fellmann et al., 2020). Some guidance regarding sharing of posthumous findings with relatives already exists in countries such as New Zealand and Japan (Branum & Wolf, 2015). In the UK, the *ABC* v St George Healthcare NHS Trust 2017 case in which a daughter claimed negligence due to clinicians' non‐disclosure of her father's diagnosis of Huntington's disease has been considered a step towards recognizing a relative's “right to know” with respect to genetic findings (Gordon & Koenig, 2022).

For studies where genetic data are involved, genetic counselors may be crucial in supporting communication with families after the participant's death, bearing in mind previously stated disclosure preferences, potential health implications for relatives, as well as their “right not to know” (Bak et al., 2021; Bombard et al., 2019). Further training on such communication with grieving families may need to be considered.

### 4.1 Limitations

The limitations of this review were similar to those in the previous review (Bak et al., 2020). The number of studies included is relatively small. As the search excluded non‐English articles, it produced studies mainly from North America and Europe, presenting a predominantly, though not exclusively, Western cultural perspective. Two out of the seven articles included in this review were carried out by the Bak et al. research group (Bak et al., 2021, 2023), and the perspective of their research on the issue has likely influenced the findings and conclusions of this review. The comparability of studies included in this review is limited due to a variety of methods and settings; however, this has the advantage of providing a range of perspectives and insights from varying contexts. This also applies to comparisons made between this update and the previous systematic review. Furthermore, participants that chose to take part in the included studies are likely to have favorable attitudes to research. Two included studies comprised participants who already shared their genetic and health data for research, indicating existing positive attitudes towards data sharing (Bak et al., 2021; Pacyna et al., 2020). However, given the focus on the use of such data in research, the perspectives of current research participants are also highly informative and relevant.

### 4.2 Implications

While this remains a relatively under‐explored area, the systematic review by Bak et al., and this updated review provide the evidence to date elucidating perspectives of relevant parties regarding sharing and controlling participant health and genetic data post‐mortem. Although post‐mortem data sharing seem to be generally supported by interest‐holders, the ethical guidance regarding many aspects of posthumous use of data is limited. Legislation and guidance regarding post‐mortem privacy is also sparse and contradictory (Bak et al., 2020, 2023; Etheredge, 2021; Hänold et al., 2017; Harbinja, 2013; Smolensky, 2009; Ursin & Stuifbergen, 2018). As new technology brings increasing risks to privacy, it is prudent to address these legislation gaps. It is important that this is done in a coordinated manner to facilitate international research collaborations and decrease the burdens that diverging approaches may have on research (Bak et al., 2023). Given the sensitivity of the issue, further engagement with interest‐holders is likely needed when developing guidance and policy to help resolve potentially contrasting perspectives in a transparent manner.

Both systematic reviews indicated that participants and next‐of‐kin strongly support disclosure of genetic incidental findings (Bak et al., 2020, 2021). Most participants seem willing to share this data with relatives, but mixed opinions exist regarding the ability to override the preferences of the deceased to inform surviving relatives of clinical findings (Bak et al., 2021). If current policies allow the preferences of the deceased to be overridden, this needs to be clearly disclosed to participants at the time of consent (Bak et al., 2020, 2023; Bombard et al., 2019). Given the lack of consensus, future research could explore the perspectives of researchers and experts regarding the return of post‐mortem genetic incidental findings. As genetic counselors can act as a bridge between participants and researchers, it may be beneficial to examine views regarding a system that could support clinicians with disclosure of post‐mortem incidental findings within a research context (Bak et al., 2020, 2023; Bombard et al., 2019).

This review indicates that broad consent seems to be generally accepted by interest‐holders. However, it has also revealed evidence in support of the suitability of dynamic and meta consent, especially for long‐term studies due to the likelihood that participants' preferences may change over time. As pointed out by Wiertz and Boldt, the appropriate mode of consent for studies will likely vary between jurisdictions depending on the legal protections afforded to participants, digital literacy of the public, and existing social inequalities (Wiertz & Boldt, 2022). While considerations of a suitable mode of consent are important on a study‐to‐study basis, arguably, the bigger picture that needs to be addressed are existing inequalities and legislative divergence between countries (Alrabadi et al., 2019; Bak et al., 2021, 2023; Pacyna et al., 2020).

The studies included in this systematic review also revealed differences between the ante‐mortem and post‐mortem willingness to share different types of data, but the factors that contribute to these differences have not been explored (Moulaei et al., 2023; Seltzer et al., 2019). Further qualitative research could explore the reasons behind this.

## 5 CONCLUSION

This systematic review has helped to elucidate further contemporary interest‐holder perspectives on the acceptability of sharing and re‐using health and genetic data post‐mortem, respecting the privacy of deceased individuals and the appropriate modes of consent in research that uses decedents' data. These issues need to be urgently addressed given the increasing use of biobanks and the emergence of AI and big‐data technologies that are presenting new privacy risks for the living and decedents.

## AUTHOR CONTRIBUTIONS

All authors contributed to the screening of studies as well as extraction and interpretation of data. The results and final version of the manuscript were reviewed and approved by all authors.

## FUNDING INFORMATION

No financial assistance was received in support of this study.

## CONFLICT OF INTEREST STATEMENT

Katarzyna Kucharska was employed by Besins Healthcare during a part of the completion of this systematic review. Dr. Ahmed Hassan Ali and Dr. Frank Moriarty declare no conflicts of interest.

## ETHICS STATEMENT

Human studies and informed consent: Ethical approval and informed consent were not required because this review was based only on published literature and has not involved any interventions or data collection from human or animal subjects.

## Supporting information


Table S1

Table S2

Table S3

## Data Availability

All data supporting the findings of this review were extracted from previously published articles and are presented within the main body of the manuscript and the [Link], [Link], [Link].

## References

[jgc470186-bib-0001] Alrabadi, N. , Makhlouf, H. , Khabour, O. F. , & Alzoubi, K. H. (2019). Jordanians' perspectives on open consent in biomedical research. Risk Management and Healthcare Policy, 12, 265–273. 10.2147/rmhp.S217209 31819687 PMC6897061

[jgc470186-bib-0002] Bak, M. A. R. , Ploem, M. C. , Atesyürek, H. , Blom, M. T. , Tan, H. L. , & Willems, D. L. (2020). Stakeholders' perspectives on the post‐mortem use of genetic and health‐related data for research: A systematic review. European Journal of Human Genetics, 28(4), 403–416. 10.1038/s41431-019-0503-5 31527854 PMC7080773

[jgc470186-bib-0003] Bak, M. A. R. , Veeken, R. , Blom, M. T. , Tan, H. L. , & Willems, D. L. (2021). Health data research on sudden cardiac arrest: Perspectives of survivors and their next‐of‐kin. BMC Medical Ethics, 22(1), 7. 10.1186/s12910-021-00576-9 33509184 PMC7844916

[jgc470186-bib-0004] Bak, M. A. R. , Vroonland, J. C. H. , Blom, M. T. , Damjanovic, D. , Willems, D. L. , Tan, H. L. , & Corrette Ploem, M. (2023). Data‐driven sudden cardiac arrest research in Europe: Experts' perspectives on ethical challenges and governance strategies. Resuscitation Plus, 15, 100414. 10.1016/j.resplu.2023.100414 37363125 PMC10285638

[jgc470186-bib-0005] Bak, M. A. R. , & Willems, D. L. (2022). Contextual exceptionalism after death: An information ethics approach to post‐mortem privacy in health data research. Science and Engineering Ethics, 28(4), 32. 10.1007/s11948-022-00387-0 35922650 PMC9349167

[jgc470186-bib-0006] Bombard, Y. , Brothers, K. B. , Fitzgerald‐Butt, S. , Garrison, N. A. , Jamal, L. , James, C. A. , Jarvik, G. P. , McCormick, J. B. , Nelson, T. N. , Ormond, K. E. , Rehm, H. L. , Richer, J. , Souzeau, E. , Vassy, J. L. , Wagner, J. K. , & Levy, H. P. (2019). The responsibility to recontact research participants after reinterpretation of genetic and genomic research results. American Journal of Human Genetics, 104(4), 578–595. 10.1016/j.ajhg.2019.02.025 30951675 PMC6451731

[jgc470186-bib-0007] Branum, R. , & Wolf, S. M. (2015). International policies on sharing genomic research results with relatives: Approaches to balancing privacy with access. The Journal of Law, Medicine & Ethics, 43(3), 576–593. 10.1111/jlme.12301 PMC4617202 26479568

[jgc470186-bib-0008] Carmi, S. (2020). The challenges of maintaining genetic privacy. eLife, 9, e54467. 10.7554/eLife.54467 31908269 PMC6946563

[jgc470186-bib-0009] de Man, Y. , Wieland‐Jorna, Y. , Torensma, B. , de Wit, K. , Francke, A. L. , Oosterveld‐Vlug, M. G. , & Verheij, R. A. (2023). Opt‐in and opt‐out consent procedures for the reuse of routinely recorded health data in scientific research and their consequences for consent rate and consent bias: Systematic review. Journal of Medical Internet Research, 25, e42131. 10.2196/42131 36853745 PMC10015347

[jgc470186-bib-0010] Etheredge, H. R. (2021). Assessing global organ donation policies: Opt‐in vs opt‐out. Risk Management and Healthcare Policy, 14, 1985–1998. 10.2147/RMHP.S270234 34012308 PMC8128443

[jgc470186-bib-0011] Fabry, R. E. , & Alfano, M. (2024). The affective scaffolding of grief in the digital age: The case of Deathbots. Topoi, 43, 757–769. 10.1007/s11245-023-09995-2

[jgc470186-bib-0012] Fellmann, F. , Rial‐Sebbag, E. , Patch, C. , Hentze, S. , Stefandottir, V. , Mendes, Á. , van El, C. G. , Cornel, M. C. , & Forzano, F. (2020). ESHG PPPC comments on postmortem use of genetic data for research purposes. European Journal of Human Genetics, 28(2), 144–146. 10.1038/s41431-019-0525-z 31595045 PMC6974591

[jgc470186-bib-0013] Garner, P. , Hopewell, S. , Chandler, J. , MacLehose, H. , Schünemann, H. J. , Akl, E. A. , Beyene, J. , Chang, S. , Churchill, R. , Dearness, K. , Guyatt, G. , Lefebvre, C. , Liles, B. , Marshall, R. , Martínez García, L. , Mavergames, C. , Nasser, M. , Qaseem, A. , Sampson, M. , & Schünemann, H. J. (2016). When and how to update systematic reviews: Consensus and checklist. BMJ, 354, i3507. 10.1136/bmj.i3507 27443385 PMC4955793

[jgc470186-bib-0014] Gerdon, F. , Nissenbaum, H. , Bach, R. , Kreuter, F. , & Zins, S. (2021). Individual acceptance of using health data for private and public benefit: Changes during the COVID‐19 pandemic. *Harvard Data Science Review*. 10.1162/99608f92.edf2fc97

[jgc470186-bib-0015] Gordon, D. R. , & Koenig, B. A. (2022). “If relatives inherited the gene, they should inherit the data.” bringing the family into the room where bioethics happens. New Genetics and Society, 41(1), 23–46. 10.1080/14636778.2021.2007065 36090688 PMC9454889

[jgc470186-bib-0016] Gordon, D. R. , Radecki Breitkopf, C. , Robinson, M. , Petersen, W. O. , Egginton, J. S. , Chaffee, K. G. , Petersen, G. M. , Wolf, S. M. , & Koenig, B. A. (2019). Should researchers offer results to family members of cancer biobank participants? A mixed‐methods study of proband and family preferences. AJOB Empirical Bioethics, 10(1), 1–22. 10.1080/23294515.2018.1546241 30596322 PMC6443426

[jgc470186-bib-0017] Hänold, S. , Forgó, N. , Kobeissi, D. , & Nwankwo, I. (2017). Legal perspectives on post‐mortem use of biomaterial and data for research: A focus on the German situation. European Journal of Health Law, 24(3), 311–327. 10.1163/15718093-12341415

[jgc470186-bib-0018] Harbinja, E. (2013). Does the EU data protection regime protect post‐mortem privacy and what could be the potential alternatives? SCRIPTed, 10(1), 19–38.

[jgc470186-bib-0019] Harbinja, E. , & Pearce, H. (2020). Your data will never die, but you will: A comparative analysis of US and UK post‐mortem data donation frameworks. Computer Law and Security Review, 36, 105403. 10.1016/j.clsr.2020.105403

[jgc470186-bib-0020] Higgins, J. , Thomas, J. , Chandler, J. , Cumpston, M. , Li, T. , Page, M. , & Welch, V. (2023). Cochrane handbook for systematic reviews of interventions version 6.4 . 10.1002/14651858.ED000142 PMC10284251 31643080

[jgc470186-bib-0021] Ho, A. , Leach, E. , Virani, A. , Arbour, L. , Bartels, K. , & Wong, E. K. (2022). Cascade testing for inherited arrhythmia conditions: Experiences and attitudes of family communication approaches for a Canadian cohort. Journal of Genetic Counseling, 31(3), 815–828. 10.1002/jgc4.1550 35032083

[jgc470186-bib-0022] Joanna Briggs Institute Critical Appraisal Tools . https://jbi.global/critical‐appraisal‐tools

[jgc470186-bib-0023] Krutzinna, J. , Taddeo, M. , & Floridi, L. (2019). Enabling posthumous medical data donation: An appeal for the ethical utilisation of personal health data. Science and Engineering Ethics, 25(5), 1357–1387. 10.1007/s11948-018-0067-8 30357557

[jgc470186-bib-0024] Kucharska, K. , Hassan Ali, A. , & Moriarty, F. (2024). An updated systematic review exploring the perspectives of stakeholders on the post‐mortem use of health and genetic data for research; CRD42024531729 . https://www.crd.york.ac.uk/prospero/display_record.php?ID=CRD42024531729

[jgc470186-bib-0025] Lessard, D. , Dubé, K. , Bilodeau, M. , Keeler, P. , Margolese, S. , Rosenes, R. , Sinyavskaya, L. , Durand, M. , Benko, E. , Kovacs, C. , Guerlotté, C. , Tharao, W. , Arnold, K. , Masching, R. , Taylor, D. , Sousa, J. , Ostrowski, M. , Taylor, J. , Kaytes, A. , … Costiniuk, C. T. (2022). Willingness of older Canadians with HIV to participate in HIV cure research near and after the end of life: A mixed‐method study. AIDS Research and Human Retroviruses, 38(8), 670–682. 10.1089/aid.2022.0006 35778845 PMC9483839

[jgc470186-bib-0026] McGrath, E. R. , Kirby, N. , & Shiely, F. (2024). Use of participant data and biological samples is insufficiently described in participant information leaflets. Journal of Clinical Epidemiology, 177, 111590. 10.1016/j.jclinepi.2024.111590 39505053

[jgc470186-bib-0027] Moulaei, K. , Iranmanesh, E. , Amiri, P. , & Ahmadian, L. (2023). Attitudes of Covid‐19 patients toward sharing their health data: A survey‐based study to understand security and privacy concerns. Health Science Reports, 6(3), e1132. 10.1002/hsr2.1132 36865528 PMC9971706

[jgc470186-bib-0028] Nuffield Council on Bioethics . (2015). The collection, linking and use of data in biomedical research and healthcare: Ethical issues. Nuffield Council on Bioethics.

[jgc470186-bib-0029] Nuffield Council on Bioethics . (2023). DNA.I. – Early findings and emerging questions on the use of AI in genomics. Nuffield Council on Bioethics.

[jgc470186-bib-0030] OECD . (2009). Guidelines on human biobanks and genetic research databases. OECD. 20443450

[jgc470186-bib-0031] Pacyna, J. E. , McCormick, J. B. , Olson, J. E. , Winkler, E. M. , Bublitz, J. T. , Hathcock, M. A. , & Sharp, R. R. (2020). Assessing the stability of biobank donor preferences regarding sample use: Evidence supporting the value of dynamic consent. European Journal of Human Genetics, 28(9), 1168–1177. 10.1038/s41431-020-0625-9 32327712 PMC7608348

[jgc470186-bib-0032] Page, M. J. , McKenzie, J. E. , Bossuyt, P. M. , Boutron, I. , Hoffmann, T. C. , Mulrow, C. D. , Shamseer, L. , Tetzlaff, J. M. , Akl, E. A. , Brennan, S. E. , Chou, R. , Glanville, J. , Grimshaw, J. M. , Hróbjartsson, A. , Lalu, M. M. , Li, T. , Loder, E. W. , Mayo‐Wilson, E. , McDonald, S. , & Moher, D. (2021). The PRISMA 2020 statement: An updated guideline for reporting systematic reviews. BMJ, 372, n71. 10.1136/bmj.n71 33782057 PMC8005924

[jgc470186-bib-0033] Pearce, H. (2022). Our data? An examination of the possible role of individual consent in the regulation of posthumous medical data donation (PMDD). Computer Law and Security Review, 45, 105663. 10.1016/j.clsr.2022.105663

[jgc470186-bib-0034] Rubinger, L. , Gazendam, A. , Ekhtiari, S. , & Bhandari, M. (2023). Machine learning and artificial intelligence in research and healthcare. Injury, 54, S69–S73. 10.1016/j.injury.2022.01.046 35135685

[jgc470186-bib-0035] Seltzer, E. , Goldshear, J. , Guntuku, S. C. , Grande, D. , Asch, D. A. , Klinger, E. V. , & Merchant, R. M. (2019). Patients' willingness to share digital health and non‐health data for research: A cross‐sectional study. BMC Medical Informatics and Decision Making, 19(1), 157. 10.1186/s12911-019-0886-9 31395102 PMC6686530

[jgc470186-bib-0036] Smolensky, K. (2009). Rights of the dead. SSRN Electronic Journal. 1–34. 10.2139/ssrn.924499

[jgc470186-bib-0037] Sweeney L. , Abu A. , & Winn J. (2013) Identifying Participants in the Personal Genome Project by Name. Harvard University Data Privacy Lab. Report number 1021‐1

[jgc470186-bib-0038] Terletska, M. (2023). Post‐mortem privacy and the latest GDPR legislative shifts .

[jgc470186-bib-0039] Ursin, L. , & Stuifbergen, M. (2018). Ethics of dead participants: Policy recommendations for biobank research. Journal of Medical Ethics, 44(10), 695–699. 10.1136/medethics-2017-104241 29921618

[jgc470186-bib-0040] Wiertz, S. , & Boldt, J. (2022). Evaluating models of consent in changing health research environments. Medicine, Health Care, and Philosophy, 25(2), 269–280. 10.1007/s11019-022-10074-3 35286521 PMC9135890

[jgc470186-bib-0041] Wolf, S. M. , Branum, R. , Koenig, B. A. , Petersen, G. M. , Berry, S. A. , Beskow, L. M. , Daly, M. B. , Fernandez, C. V. , Green, R. C. , LeRoy, B. S. , Lindor, N. M. , O'Rourke, P. P. , Breitkopf, C. R. , Rothstein, M. A. , Van Ness, B. , & Wilfond, B. S. (2015). Returning a research Participant's genomic results to relatives: Analysis and recommendations. The Journal of Law, Medicine & Ethics, 43(3), 440–463. 10.1111/jlme.12288 PMC4617203 26479555

