# Supplementary material for: Exploring perspectives of interest‐holders on the use of health and genomic data from deceased participants in research: An updated systematic review
Source: J Genet Couns. 2026 Mar 2;35(2):e70186. doi: 10.1002/jgc4.70186 (PMC12954163; doi:10.1002/jgc4.70186)
Supplement: Supplementary file 1 — Table S1 [file JGC4-35-0-s003.docx]

| **Set#** | **Search string** | **Results** |
| --- | --- | --- |
| 1 | ((MH "Registries") OR "health data" OR "health information" OR (MH "Electronic Health Records") OR (TI (biobank* OR bio-bank* OR biorepository*) OR AB (biobank* OR bio-bank* OR biorepository*)) OR (MH "Databases, Factual") OR (TI (registry OR registries OR databank* OR genomic* OR "genetic research" OR genetic* OR "individual finding*" OR "genetic finding*") OR (AB registry OR registries OR databank* OR genomic* OR "genetic research" OR genetic* OR "individual finding*" OR "genetic finding* )) OR (MH "Genetics")) | 365,995 |
| 2 | ((MH "Privacy") OR (MH "Confidentiality") OR (MH "Personally Identifiable Information") OR (TI (privacy* OR confidential*) OR AB (privacy* OR confidential*)) OR (MH "Informed Consent") OR (TI ( "informed consent") OR AB ("informed consent")) OR (MH "Ethics, Research") OR (MH "Patient Rights") OR (TI (disclos*) OR AB (disclos*)) OR (MH "Information Dissemination") OR (MH "Communication") OR (TI (communicat*) OR AB ((communicat*)) OR (MH "Duty to Recontact")) | 265,158 |
| 3 | ((MH "Patients") OR (MH "Stakeholder Participation") OR (TI (famil* OR relative* OR participant* OR population OR public OR community OR societ*) OR AB (famil* OR relative* OR participant* OR population OR public OR community OR societ*)) OR (MH "Research Subjects") OR (TI (researchers OR institutions) OR AB (researchers OR institutions)) | 1,853,156 |
| 4 | ((MH "Death") OR (TI (deceased OR death* OR departed OR died OR dead OR "post-mortem" OR postmortem OR posthumous) OR AB (deceased OR death* OR departed OR died OR dead OR "post-mortem" OR postmortem OR posthumous)) | 274,220 |
| 5 | TI (opinions OR perspectives OR views OR experiences OR viewpoint* OR willingness OR preference* OR attitude* OR impact OR choice* OR support) OR AB (opinions OR perspectives OR views OR experiences OR viewpoint* OR willingness OR preference* OR attitude* OR impact OR choice* OR support) | 1,480,884 |
| 6 | (#1 AND #2 AND #3 AND #4 AND #5) Narrow by Language: - english Limiters - Publication Date: 20190101-20240329 | 79 |
