# Supplementary material for: Exploring perspectives of interest‐holders on the use of health and genomic data from deceased participants in research: An updated systematic review
Source: J Genet Couns. 2026 Mar 2;35(2):e70186. doi: 10.1002/jgc4.70186 (PMC12954163; doi:10.1002/jgc4.70186)
Supplement: Supplementary file 2 — Table S2 [file JGC4-35-0-s002.docx]

| **Set#** | **Search string** | **Results** |
| --- | --- | --- |
| 1 | TS=”health data” OR TS=”health information” OR TS=biobank* OR TS=bio-bank* OR TS=biospecimen* OR TS=biorepository* OR TS=registry OR TS=registries OR TS=databank* OR TS=”big data” OR TS=genomic* OR TS=genetic | 2,256,467 |
| 2 | TS=opinions OR TS=perspective* OR TS=views OR TS=viewpoint* OR TS=motivation* OR TS=willingness OR TS=preference* OR TS=attitude* OR TS=impact OR TS=choice* OR TS=experiences OR TS=support | 12,650,432 |
| 3 | TS=participant* OR TS=population OR TS=public OR TS=community OR TS=societ* OR TS=patient* OR TS=famil* OR TS=relative* OR TS=researchers OR TS=institutions | 18,552,243 |
| 4 | TS=privacy* OR TS=confidential* OR TS=informed consent OR TS=communicat* | 1,611,271 |
| 5 | TS=deceased OR TS=death* OR TS=departed OR TS=died OR TS=dead OR TS=post-mortem OR TS=postmortem OR TS=posthumous | 1,839,578 |
| 6 | (#1 AND #2 AND #3 AND #4 AND #5) AND English (Languages) and Article (Document Types) | 252 |
