# Supplementary material for: Exploring perspectives of interest‐holders on the use of health and genomic data from deceased participants in research: An updated systematic review
Source: J Genet Couns. 2026 Mar 2;35(2):e70186. doi: 10.1002/jgc4.70186 (PMC12954163; doi:10.1002/jgc4.70186)
Supplement: Supplementary file 3 — Table S3 [file JGC4-35-0-s001.docx]

| **Set#** | **Search string** | **Results** |
| --- | --- | --- |
| 1 | ('health data':ti,ab OR 'health information':ti,ab OR biobank*:ti,ab OR 'bio bank*':ti,ab OR biospecimen*:ti,ab OR biorepository*:ti,ab OR registry:ti,ab OR registries:ti,ab OR databank*:ti,ab OR 'big data':ti,ab OR genomic*:ti,ab OR genetic*:ti,ab) | 2,302,436 |
| 2 | (opinions:ti,ab OR perspective*:ti,ab OR views:ti,ab OR viewpoint*:ti,ab OR motivation*:ti,ab OR willingness:ti,ab OR preference*:ti,ab OR attitude*:ti,ab OR impact:ti,ab OR choice*:ti,ab OR experiences:ti,ab OR support:ti,ab) | 5,268,110 |
| 3 | (participant*:ti,ab OR population:ti,ab OR public:ti,ab OR community:ti,ab OR societ*:ti,ab OR patient*:ti,ab OR famil*:ti,ab OR relative*:ti,ab OR researchers:ti,ab OR institutions:ti,ab) | 18,283,202 |
| 4 | (deceased:ti,ab OR death*:ti,ab OR departed:ti,ab OR died:ti,ab OR dead:ti,ab OR 'post mortem':ti,ab OR postmortem:ti,ab OR posthumous:ti,ab) | 2,009,600 |
| 5 | (privacy*:ti,ab OR confidential*:ti,ab OR (informed AND consent:ti,ab) OR communicat*:ti,ab) | 686,984 |
| 6 | #1 AND #2 AND #3 AND #4 AND #5 AND [article]/lim AND [2019-2024]/py AND [english]/lim | 123 |
